# Psychological stress and functional ovarian suppression in women with PCOM: an observational study of FHA-like neuroendocrine phenotypes

**DOI:** 10.1007/s00737-025-01657-z

**Published:** 2026-01-07

**Authors:** Vanessa Silva, Sérgio Soares, Rui Miguelote

**Affiliations:** 1 Medically Assisted Reproduction Center, Gynecology and Obstetrics Service - Local Health Unit of Alto Ave, Guimarães, Portugal; 2 School of Medicine, University of Minho, Braga, Portugal; 3 Reproductive Medicine, IVI RMA, Lisbon, Portugal

**Keywords:** Psychological stress, Polycystic ovarian morphology, LH/FSH ratio, AMH, Leptin

## Abstract

**Propose:**

To examine how chronic psychological stress alters gonadotropin dynamics and disrupts ovarian endocrine function in women with polycystic ovarian morphology (PCOM), and to discuss the modulatory role of leptin in this process.

**Methods:**

In this cross-sectional study of 134 women, participants were classified into four groups: three subgroups of women with oligomenorrhea—PCOM with stress, PCOM without stress, and NON-PCOM/NON-STRESS—and a comparison group of eumenorrheic controls. Psychological stress was assessed with validated psychometric instruments (STAI, HADS, PSS-10), and a composite Stress Index was derived. PCOM was defined according to the 2023 International Evidence-based Guideline for PCOS. Stress status was classified using established cut-offs for each instrument, with non-stress cohorts defined by scores consistently below clinical thresholds. Hormonal profiling included LH, FSH, estradiol, AMH, leptin, cortisol, and ACTH. Mediation and moderation models were employed to examine the relationships among stress, leptin, the LH/FSH ratio, and ovarian endocrine markers, as AMH and estradiol.

**Results:**

Women in the PCOM–STRESS group exhibited significantly lower LH levels, LH/FSH ratios, and AMH concentrations compared to PCOM–NON–STRESS, despite similar ovarian morphology and preserved FSH levels. Mediation analysis revealed that the LH/FSH ratio significantly mediated the effect of psychological stress on both estradiol and AMH levels. Moderation analysis indicated that leptin modulated the impact of stress on the LH/FSH ratio (interaction p = 0.004), with more pronounced suppressive effects of psychological stress under low leptin levels. Despite high psychological stress, women in the PCOM–STRESS group showed no activation of the HPA axis, suggesting neuroendocrine resilience or adaptation. These findings highlight the clinical value of assessing both psychological and metabolic context in women with ambiguous ovulatory dysfunction.

**Conclusion:**

Chronic psychological stress in women with PCOM is associated with functional suppression of LH and ovarian endocrine output, reflecting an attenuation of the typical PCOS endocrine phenotype despite the polycystic ovarian morphology. Leptin modulates individual susceptibility to stress-induced reproductive suppression, acting as a potential permissive signal of hypothalamic resilience. Assessing gonadotropin ratios and metabolic context may improve diagnostic accuracy in women with ambiguous ovulatory dysfunction.

**Supplementary Information:**

The online version contains supplementary material available at 10.1007/s00737-025-01657-z.

## Article highlights

PCOM with chronic stress shows functional ovarian suppression despite preserved morphology.

Stress suppresses LH-dependent AMH and estradiol output, indicating impaired granulosa and theca cell activity despite preserved PCOM.

Leptin modulates reproductive sensitivity to stress, especially at low concentrations.

PCOM–STRESS profiles align more with FHA than with classical PCOS.

Leptin–stress interaction supports differentiation between FHA-like and PCOS-like PCOM presentations.

**Supplementary Information:**

The online version contains supplementary material available at 10.1007/s00737-025-01657-z.

## Introduction

Polycystic ovarian morphology (PCOM) is defined in the 2023 international guideline as the presence of ≥ 20 follicles measuring 2–9 mm in at least one ovary and/or an ovarian volume ≥ 10 cm³ on modern ultrasound (Teede et al. 2023). Although recognized as one of the three Rotterdam diagnostic criteria for polycystic ovary syndrome (PCOS), PCOM itself represents a morphological finding rather than a diagnosis. PCOS, in contrast, is a clinical syndrome requiring the integration of morphological, endocrine, and metabolic features. Distinguishing between PCOM and PCOS is clinically significant, as PCOM is also found in healthy women and in those with functional hypothalamic amenorrhea (FHA), highlighting its limited specificity and broad phenotypic spectrum (Ott 2025). Emerging evidence suggests that PCOM spans a continuum of phenotypes shaped not only by intrinsic ovarian factors but also by external modulators such as chronic psychological stress (Hager et al. 2023; Phylactou et al. 2021).

In women with oligo/amenorrhea (OA) and PCOM, unrecognized stress can further blur the diagnostic boundaries between PCOS and FHA (Ott 2025). Identical ovarian morphology may mask distinct mechanisms of dysfunction (Phylactou et al., 2021; Wang and Lobo, 2008), and stress-related PCOM remains invisible in clinical practice, mainly due to its stress-related nature. In FHA, stress suppresses GnRH pulsatility, leading to reduced LH secretion, impaired gonadotropin-dependent ovarian function, and ovulatory arrest (Gordon et al., 2017; McCosh et al., 2019). Prolonged activation of the hypothalamic–pituitary–adrenal (HPA) axis further reinforces this suppression, also disrupting hypothalamic–pituitary–gonadal signaling and compromising ovarian function (Valsamakis et al., 2019; Saadedine et al., 2025). As a result, women with PCOM may be misclassified as having PCOS when their ovulatory dysfunction actually reflects FHA-like hypothalamic inhibition. This highlights the importance of complementing morphological criteria with functional and psychosocial assessments.

Leptin, an adipokine linking energy availability to reproduction, modulates this neuroendocrine network by influencing gonadotropin secretion and granulosa cell activity (Evans et al., 2022). Evidence suggests that leptin may regulate anti-Müllerian hormone (AMH) production directly or indirectly through its interaction with LH signaling (Merhi et al., 2013). Yet, the impact of chronic psychological stress on leptin signaling and ovarian endocrine function in women with PCOM remains poorly understood.

This study aimed to investigate how psychological stress alters gonadotropin secretion and ovarian endocrine responsiveness in women with OA and PCOM, and to examine the potential modulatory role of leptin in this context. By integrating hormonal, psychological, and metabolic assessments, we sought to clarify stress-related patterns of hypothalamic–pituitary–ovarian disruption that may help explain the clinical and hormonal overlap between PCOS and FHA.

## Materials and methods

This observational cross-sectional study was conducted in northern Portugal between January 2020 and August 2022. Participants were recruited from the general population through online platforms and local media. Women with regular or irregular menstrual cycles were invited to undergo a comprehensive hormonal and psychological evaluation to explore the causes of anovulation, including stress-related and polycystic ovarian mechanisms. Interested individuals completed an online questionnaire addressing sociodemographic and clinical variables, menstrual characteristics, and psychological stress. All participants had experienced spontaneous menarche at least two years before enrollment. Regular menstrual cycles were defined as 21–35 days in length, and OA as an average cycle length > 35 days over the past year (Teede et al. 2023). Women with OA were stratified according to the Rotterdam criteria for PCOM and further categorized by psychological stress burden, using validated psychometric cut-offs described below (Teede et al. 2023). 

Exclusion criteria included age ≥ 40 years, pregnancy, lactation, thyroid dysfunction, hyperprolactinemia, and recent use of medications interfering with endocrine function or psychotropic drugs. Women with FSH > 18 mIU/mL, AMH < 0.5 ng/mL or age ≥ 40 years were excluded to avoid the confounding effect of diminished ovarian reserve. Women with a history of eating disorders, recent caloric restriction, high-performance athletic training, or significant weight loss (> 10% BMI) were excluded due to their known association with FHA-like profiles. These exclusions allowed us to focus on OA–PCOM presentations with less obvious causes, enriching the cohort for stress-related phenotypes that are harder to recognize clinically and therefore more prone to misclassification as PCOS.

A total of 200 women were initially screened. Of these, 52 were excluded (38 for not meeting inclusion criteria, including use of hormonal contraception, endocrine disorders, or poor ovarian reserve; and 14 due to incomplete baseline data or refusal to participate), leaving 148 women eligible for enrollment. After enrollment, 14 were excluded due to withdrawal of consent (*n* = 6), missing blood samples (*n* = 4), or incomplete ultrasound/psychometric assessments (*n* = 4), resulting in a final analytic sample of 134 women. Psychological stress was assessed using validated Portuguese versions of the Perceived Stress Scale (PSS-10), the Hospital Anxiety and Depression Scale (HADS), and the State-Trait Anxiety Inventory (STAI-Y1 and Y2) (Zigmond and Snaith 1983; Silva et al. 2003; Spielberger 1983; Zigmond and Snalth 1983; Cohen et al. 1983; Trigo et al. 2010; Pais-Ribeiro et al. 2007). Clinically significant distress was defined as STAI-Trait ≥ 40, STAI-State ≥ 40, HADS Total ≥ 11, HADS-Anxiety ≥ 8, HADS-Depression ≥ 8, and PSS-10 ≥ 20 (Kassahun et al. 2022; Fomenko et al. 2025; Deshields et al. 2021). A Stress Index was computed by summing the HADS Total and STAI-Trait scores, reflecting chronic psychological distress as a continuous variable (Knowles and Olatunji 2020). This index is not a categorical diagnostic tool but an exploratory composite designed to integrate psychological burden, with higher scores indicating greater levels of chronic distress.

All participants underwent physical, gynecological, and anthropometric assessments (including height, weight, and BMI), as well as blood collection and a transvaginal ultrasound. Examinations were scheduled in the early follicular phase (cycle days 2–5) of a spontaneous cycle, as gonadotropin levels are stable in this window and reflect baseline ovarian endocrine activity. In women with amenorrhea, a blood sample was retrieved at any random time point. Ultrasound was performed using a standardized 2D/3D 5–9 MHz transvaginal probe (Samsung^®^ HS50). Follicles measuring 2–9 mm were counted per ovary and summed to obtain the total follicle number in both ovaries (FNBO). Ovarian volume (OV) was calculated in the absence of masses. PCOM was defined according to the 2023 International Evidence-based Guideline for PCOS as ≥ 20 follicles per ovary or OV ≥ 10 cm³ (Teede et al. 2023). The updated follicle threshold reflects the use of modern high-resolution ultrasound technology, which supersedes the earlier cutoff of 12 follicles from the 2003 Rotterdam consensus. A single experienced examiner performed all scans, and intraobserver reliability was confirmed in a random subset of participants to ensure reproducibility.

Based on menstrual pattern, ovarian morphology, and psychological stress levels, OA participants were classified into three subgroups: PCOM–STRESS, PCOM–NON-STRESS, and NON-PCOM/NON-STRESS. A fourth group comprised eumenorrheic controls.

Fasting blood samples were collected between 08:00 and 10:00 a.m. Hormonal measurements included LH, FSH, estradiol, cortisol, ACTH (chemiluminescence), leptin (radioimmunoassay – Diagnostics Biochem Canada Cat# CAN-L-4260, RRID: AB_3532206), and AMH (enzyme immunoassay).

To reflect functional hormonal relationships, three composite variables were derived: the LH/FSH ratio as a marker of gonadotropic regulation, the AMH/FNBO ratio as an index of granulosa cell output per follicle, and the Stress Index, as described above. All hormones and derived indices were subsequently grouped into three functional neuroendocrine axes: the HPG axis (LH, FSH, LH/FSH ratio, AMH, AMH/FNBO ratio), the HPA axis (ACTH, cortisol, Stress Index), and leptin together with BMI as indicators of metabolic context.

To explore physiological relevance, targeted correlations were tested between LH and the LH/FSH ratio to examine gonadotropic balance, between LH and the AMH/FNBO ratio as a proxy of LH-driven granulosa activity, and between AMH and the AMH/FNBO ratio to assess granulosa-specific efficiency.

Statistical analyses were performed using IBM SPSS Statistics (version 25.0), with an α level of 0.05. Between-group comparisons were conducted using one-way ANOVA and general linear models adjusting for covariates such as BMI, followed by post hoc Sidak or Bonferroni corrections. Categorical variables were compared using chi-square tests. Correlations were analyzed using Pearson’s or Spearman’s methods, depending on distribution.

To investigate how psychological stress might influence estradiol and AMH in the PCOM–STRESS subgroup, we tested mediation and moderation models with multiple linear regression. In both models, the LH/FSH ratio was entered as a potential mediator between stress and either estradiol or AMH. Moderation and mediation models were tested using PROCESS v4.2 for SPSS, with bias-corrected and accelerated (bCa) bootstrapped confidence intervals (5,000 resamples). Model assumptions were evaluated by testing normality of distributions, variance inflation factors for multicollinearity, and residual plots for homoscedasticity. In a second step, moderation was examined by adding a Stress × Leptin interaction term to the regression predicting the LH/FSH ratio, with predictors mean-centered before computing interaction terms. Finally, a multivariable regression including LH/FSH ratio, leptin, Stress Index, BMI, and the Stress × Leptin interaction was estimated to assess their independent contributions to AMH and estradiol levels. Supplementary tables provide descriptive and correlation data. Ethical approval was obtained from the ethics committees of Hospital de Senhora da Oliveira and Hospital de Braga (reference numbers 9/2019 and 29/2018, respectively). All participants provided written informed consent, and no financial compensation was provided for participation.

## Results

A total of 134 women were allocated to four groups: PCOM–NON-STRESS (*n* = 20), PCOM–STRESS (*n* = 45), NON-PCOM/STRESS (*n* = 26), and controls (*n* = 43). To account for unequal group sizes, all analyses used bootstrapped confidence intervals, with controls as the reference. Subgroup classification combined categorical psychometric cut-offs with ovarian morphology, whereas regression models incorporated the continuous Stress Index (see Methods). Results are presented across three domains: the HPG axis (gonadotropic and granulosa cell function), the HPA axis (stress hormones), and the metabolic context (leptin and BMI). For clarity, Table 2 reports only significant correlations (*p* < 0.05) and trends (0.05–0.10); the complete set of tested associations appears in Supplementary Table S1. Effect sizes and bootstrapped 95% confidence intervals are provided in Supplementary Table S2, with a summary of key mediation and moderation pathways in Supplementary Table S2a.

### HPG axis: gonadotropin and ovarian endocrine profiles

Despite similar ovarian morphology in the first three subgroups, LH concentrations, LH/FSH ratio, and AMH levels followed a stepwise gradient across the four subgroups -highest in PCOM–NON-STRESS, followed by PCOM–STRESS and NON-PCOM/STRESS, and lowest in controls (*p* < 0.001 for all comparisons) (Table 1). Indeed, in the PCOM–STRESS subgroup, AMH was reduced by ~ 1.5 ng/mL (95% CI − 2.3 to − 0.7) compared to PCOM–NON-STRESS, while estradiol was lower by an estimated 30.7 pg/mL (95% CI − 40.2 to − 21.1), magnitudes consistent with clinically meaningful thresholds. On the other hand, LH/FSH ratios in PCOM–NON-STRESS exceeded 1.5, a threshold often applied clinically to define hypergonadotropic PCOS profiles. Additionally, FSH levels did not differ significantly between groups (*p* = 0.205). The AMH/FNBO ratio was similar across PCOM subgroups and NON-PCOM/STRESS, but lower in controls (Table 1). In PCOM–STRESS, AMH correlated positively with LH (*r* = 0.444, *p* = 0.002) and with the LH/FSH ratio (*r* = 0.478, *p* < 0.001), while leptin also correlated with AMH (*r* = 0.343, *p* = 0.021). LH and the LH/FSH ratio were strongly correlated across all groups (*r* = 0.867–0.942, *p* < 0.001). Other associations are provided in Table 2.

**Table 1:** Differential hormonal and metabolic profiles across PCOM phenotypes under varying psychological stress

Parameter	PCOM–NON-STRESS (*n* = 20)	PCOM–STRESS (*n* = 45)	NON-PCOM/STRESS (*n* = 26)	Controls (*n* = 43)	*p*-value
ACTH (pg/mL)	15.8 ± 10.1ᵃ	14.7 ± 9.3ᵃ	20.1 ± 12.0ᵇ	15.2 ± 9.1ᵃ	0.009
Cortisol (nmol/L)	12.4 ± 5.4ᵃ	12.8 ± 4.9ᵃ	17.1 ± 4.4ᵇ	12.2 ± 4.7ᵃ	0.004
LH (IU/L)	12.5 ± 7.9ᵃ	8.5 ± 5.6ᵇ	7.1 ± 7.1ᵇ	4.4 ± 2.0ᶜ	< 0.001
FSH (IU/L)	7.5 ± 2.0	6.8 ± 1.7	7.7 ± 2.5	7.0 ± 1.7	0.205
LH: FSH ratio	1.60 ± 0.82ᵃ	1.02 ± 0.73ᵇ	0.89 ± 0.73ᶜ	0.62 ± 0.27ᵈ	< 0.001
Estradiol (pg/mL)	86.0 ± 13.2ᵃ	48.3 ± 13.4ᵇ	41.2 ± 13.8ᵇ	55.8 ± 11.2ᶜ	< 0.001
BMI (kg/m²)	26.4 ± 6.2ᵃ	25.9 ± 5.8ᵃ	23.9 ± 5.6ᵇ	22.4 ± 4.9ᵇ	0.028
Leptin (ng/mL)	24.6 ± 12.2ᵃ	21.7 ± 13.6ᵃ	23.8 ± 14.7ᵃ	18.4 ± 11.5ᵇ	0.031
AMH (ng/mL)	12.1 ± 5.1ᵃ	9.1 ± 5.1ᵇ	4.1 ± 2.6ᶜ	2.5 ± 1.6ᵈ	< 0.001
AMH/FNBO ratio	0.199 ± 0.0756ᵃ	0.185 ± 0.0851ᵃ	0.191 ± 0.0785ᵃ	0.137 ± 0.0711ᵇ	0.005
Stress Index	87.5 ± 11.9ᵃ	106.4 ± 14.7ᵇ	91.2 ± 15.3ᶜ	78.8 ± 12.5ᵈ	< 0.001

**Table 2:** Axial correlations (HPG, HPA, metabolic) stratified by PCOM phenotype and stress exposure

Variable	PCOM–STRESS	PCOM–NON-STRESS	NON-PCOM/NON-STRESS	Controls
HPG Axis				
AMH ~ LH	*r* = 0.444, *p* = 0.002			*r* = 0.519, *p* < 0.001
AMH ~ Leptin	*r* = 0.343, *p* = 0.021			
AMH ~ LH/FSH	*r* = 0.478, *p* < 0.001		*r* = 0.398, *p* = 0.044	*r* = 0.613, *p* < 0.001
AMH ~ AMH/FNBO ratio	*r* = 0.646, *p* < 0.001	*r* = 0.749, *p* < 0.001	*r* = 0.658, *p* < 0.001	*r* = 0.589, *p* < 0.001
FSH ~ LH	*r* = 0.401, *p* = 0.006	*r* = 0.730, *p* < 0.001	*r* = 0.449, *p* = 0.021	*r* = 0.325, *p* = 0.033
LH ~ LH/FSH	*r* = 0.942, *p* = 0.001	*r* = 0.872, *p* < 0.001	*r* = 0.875, *p* = 0.001	*r* = 0.867, *p* = 0.001
LH ~ AMH/FNBO ratio	*r* = 0.316, *p* = 0.039			*r* = 0.312, *p* = 0.045
HPA Axis				
Stress Index ~ LH	*r* = − 0.330, *p* = 0.027			
Stress Index ~ LH/FSH	*r* = − 0.268, *p* = 0.075			
Cortisol ~ ACTH	*r* = 0.289, *p* = 0.045	*r* = 0.496, *p* = 0.026		*r* = 0.715, *p* = 0.001
Metabolic Context				
Leptin ~ BMI	*r* = 0.708, *p* < 0.001	*r* = 0.708, *p* < 0.001	*r* = 0.404, *p* = 0.041	*r* = 0.703, *p* < 0.001
Leptin ~ Stress Index		*r* = 0.463, *p* = 0.040		
Leptin ~ FSH			*r* = 0.481, *p* = 0.013	

In the PCOM–STRESS subgroup, two simple mediation models were tested to evaluate whether the LH/FSH ratio mediates the relationship between psychological stress (measured by the Stress Index) and either AMH or estradiol levels. In both models, the LH/FSH ratio demonstrated a statistically significant indirect effect. Specifically, higher psychological stress was associated with lower ovarian hormone output: for each one-point increase in stress scores, estradiol fell by an estimated 0.19 pg/mL (95% CI − 0.40 to − 0.04) and AMH by 0.05 ng/mL (95% CI − 0.10 to − 0.02) through an indirect pathway including the LH/FSH ratio as a mediator. In a final multivariate model, only the LH/FSH ratio remained significantly associated with both estradiol and AMH levels.

**Fig. 1:**
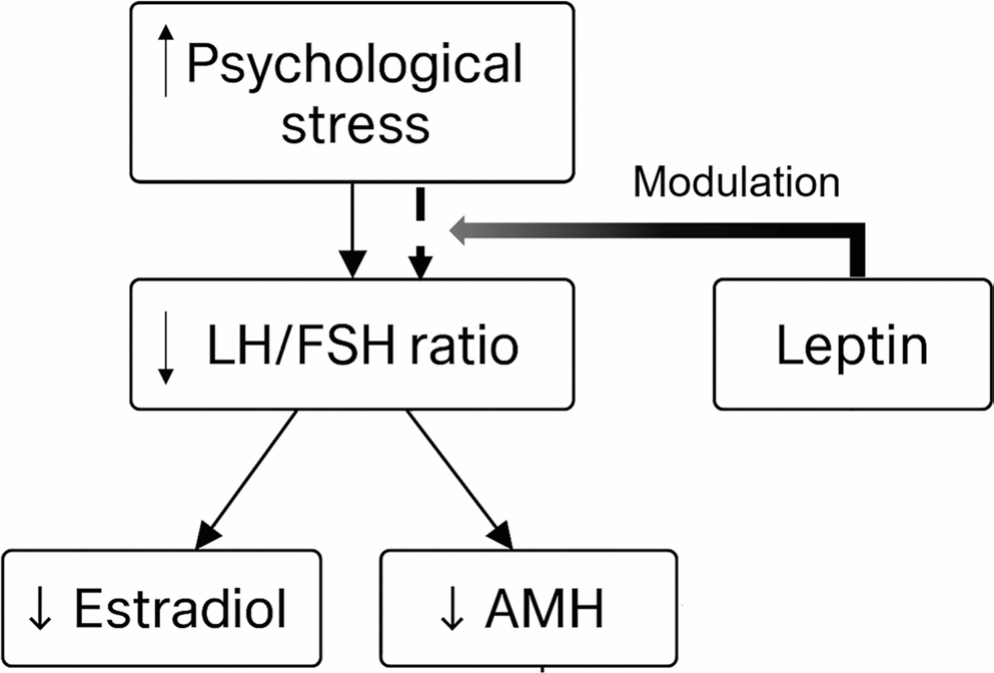
Conceptual model of stress-induced functional ovarian suppression in women with PCOM. Chronic psychological stress reduces hypothalamic GnRH pulsatility, leading to selective suppression of LH secretion and impaired granulosa and theca cell function. This disrupts the functional coupling between LH and AMH, resulting in reduced estradiol and AMH production despite the persistence of polycystic ovarian morphology. Leptin modulates the sensitivity of the reproductive axis to stress, with lower leptin levels amplifying the inhibitory effect of stress on gonadotropin dynamics. Together, these pathways describe a stress-modulated, FHA-like reproductive phenotype within the PCOM spectrum

Figure 1 illustrates the conceptual mediation model tested in the PCOM–STRESS subgroup, in which the LH/FSH ratio mediates the association between psychological stress and both AMH and estradiol levels. A concise summary of the main mediation and moderation pathways is provided in Supplementary Table S2a.

### HPA axis: psychological stress and adrenal markers

Despite elevated stress levels, morning cortisol and ACTH in PCOM–STRESS (12.8 ± 4.9; 14.7 ± 9.3) remained within normal ranges and were not significantly different from controls (12.2 ± 4.7; 15.2 ± 9.1; cortisol: *p* = 0.374; ACTH: *p* = 0.963). Cortisol and ACTH were modestly correlated in PCOM–STRESS (*r* = 0.289, *p* = 0.045), compared to stronger coupling in controls (*r* = 0.715, *p* = 0.001). In NON-PCOM/NON-STRESS, ACTH (20.1 ± 12.0 pg/mL) and cortisol (17.1 ± 4.4 nmol/L) were higher and positively correlated (*r* = 0.568, *p* = 0.004) (Tables 1 and 2).

Because psychological stress was used to subdivide the oligomenorrheic subgroups, Stress Index scores naturally differed between them, being highest in the PCOM–STRESS group, followed by NON-PCOM/STRESS, PCOM–NON-STRESS, and controls (*p* < 0.001; Table 1).

### Metabolic context: leptin and BMI effects

Controls had the lowest BMI and leptin levels (18.4 ± 11.5 ng/mL) (Tables 1 and 2). BMI and leptin were positively correlated across groups, particularly in the PCOM–STRESS group (*r* = 0.708, *p* < 0.001; Table 2). In this group, leptin was positively correlated with AMH (*r* = 0.343, *p* = 0.021) and LH/FSH (*r* = 0.274, *p* = 0.047), but not with stress.

**Fig. 2:**
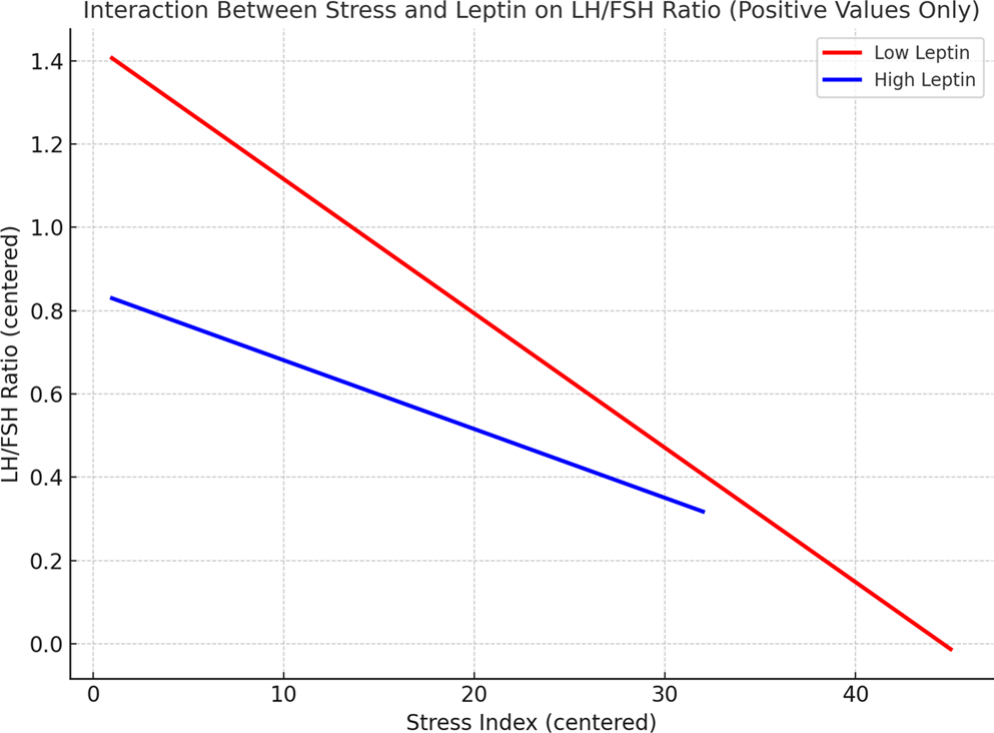
Moderation effect of leptin on the association between psychological stress and LH/FSH ratio in the PCOM-STRESS subgroup. Predicted regression lines are shown for low and high leptin groups (median split). The interaction term Stress × Leptin was statistically significant (B = − 0.001, *p* = 0.004, 95% BCa CI [–0.001, − 6.04E5]), indicating that lower leptin levels amplified the negative association between stress and gonadotropin balance

To evaluate whether leptin modulated the relationship between psychological stress and gonadotropin dynamics, a moderation model tested in PCOM–STRESS showed a significant interaction between stress and leptin in predicting the LH/FSH ratio (B = − 0.001, *p* = 0.004, 95% bCa CI [–0.001, − 6.04 × 10⁻⁵]; R² = 0.27). Lower leptin enhanced the inverse stress–LH/FSH relationship. Full results are in Supplementary Table S2; the interaction is visualized in Fig. 2.

## Overall correlation patterns (Full sample analysis)

In the full sample, AMH remained positively correlated with the LH/FSH ratio (*r* = 0.601, *p* < 0.05), and leptin correlated with the Stress Index (*r* = 0.239, *p* < 0.05). ACTH and cortisol were moderately coupled (*r* = 0.459, *p* < 0.05). The complete correlation matrix is available in Supplementary Table S1.

Values are presented as mean ± standard deviation. Superscript letters (ᵃ, ᵇ, ᶜ, ᵈ) indicate groupings based on Sidak post hoc comparisons (*p* < 0.05). Groups sharing the same letter do not differ significantly; groups with different letters differ significantly. p-values refer to overall group differences determined by one-way ANOVA.

Table 2. Within-group bivariate correlations between hormonal, stress-related, and metabolic parameters, organized by functional axis and diagnostic subgroup. Values represent Pearson’s (or Spearman’s, when appropriate) correlation coefficients (r) and their corresponding p-values. Only statistically significant correlations (*p* < 0.05) or trends (*p* < 0.10) are shown. Empty cells indicate non-significant. FNBO: Follicle Number in Both Ovaries; AMH: Anti-Müllerian Hormone; LH: Luteinizing Hormone; FSH: Follicle-Stimulating Hormone; BMI: Body Mass Index. For details on derived variables (AMH: FNBO ratio and Stress Index), see Materials and Methods.

## Discussion

### Hypothalamic suppression and ovarian dysfunction in stress-sensitive PCOM

This study suggests that chronic psychological stress contributes to ovarian dysfunction primarily through central neuroendocrine suppression, with downstream effects on granulosa and theca cells (Hu et al. 2024; Zhou et al. 2024). Elevated stress scores, measured by validated psychometric tools, were associated with reduced hypothalamic drive, reflected in lower LH secretion. Although GnRH pulsatility was not directly assessed, the observed pattern of suppressed LH and preserved FSH supports a selective reduction in GnRH pulse frequency, as described in experimental models (McCosh et al. 2019, 2022; Grachev et al. 2014; Yip et al. 2021). Consistently, the PCOM–STRESS subgroup showed lower LH concentrations and LH/FSH ratios than PCOM–NON-STRESS, while FSH remained stable—a profile indicative of stress-related hypothalamic adaptation (Saadedine et al. 2025). By combining hormonal, psychological, and metabolic assessments, we identified a stress-sensitive reproductive phenotype within PCOM (the PCOM–STRESS phenotype) in which leptin emerged as a contextual modulator of hypothalamic–pituitary sensitivity (Fig. 1).

The parallel reduction in estradiol in PCOM–STRESS suggests impaired theca cell steroidogenesis due to reduced LH signaling (Gordon et al. 2017; McCosh et al. 2019; Zhou et al. 2024). Because LH stimulates theca androgen production, its suppression creates a suboptimal paracrine environment for granulosa cells (Wang et al. 2018). Although both PCOM subgroups fulfilled diagnostic criteria Rotterdam for PCOS, stressed women had significantly lower AMH, pointing to granulosa suppression secondary to diminished LH-driven androgen supply and reduced aromatase activity (Teede et al. 2023; Yang et al. 2017; Dong et al. 2017; Carlsen et al. 2009; Ott 2025). The positive association between LH and AMH, observed predominantly in PCOM–NON-STRESS, reinforces the regulatory role of LH in granulosa-derived AMH secretion (Clemente et al. 2021).

### FHA-like suppression in PCOM: a diagnostic pitfall within PCOS criteria

Our subgrouping strategy extends the Rotterdam framework (PCOM with OA) by adding validated psychometric thresholds to detect stress-related, FHA-like suppression within PCOM. This reveals that women who technically fulfil PCOS criteria may, in fact, exhibit endocrine patterns more consistent with FHA–PCOM. Without accounting for stress burden and functional markers, such an overlap carries a high risk of misclassification (Ott 2025). We do not propose replacing established PCOS or FHA consensus definitions; instead, we argue that stress-based stratification can refine these frameworks by identifying a subset of women whose anovulation is primarily driven by hypothalamic inhibition rather than ovarian hyperfunction. Although still exploratory and requiring longitudinal validation, this approach highlights the need for diagnostic models that integrate morphology with functional assessment to prevent clinically consequential misdiagnoses.

### Altered ACTH–cortisol coupling in stress-sensitive PCOM

In the PCOM–STRESS subgroup, ACTH and cortisol levels remained within physiological ranges despite high psychological stress scores, and their correlation was weaker than in the control group. This pattern is consistent with a blunted HPA axis response, a recognized adaptation to chronic stress in which basal hormone levels appear normal but dynamic coupling becomes attenuated (Herman et al. 2016; Russell and Lightman 2019; Dalile et al. 2022; Wichmann et al. 2017; Creswell et al. 2025). Similar uncoupling has been described in PTSD, burnout, and FHA, where reduced cortisol responses to CRH stimulation indicate altered central regulation (Herman et al. 2016; Russell and Lightman 2019; Tilbrook and Clarke 2006). By contrast, the NON-PCOM/STRESS group showed elevated ACTH and cortisol, consistent with an earlier, reactive activation of the HPA axis (Herman et al. 2016). These findings suggest that PCOM–STRESS reflects a chronically adapted phenotype, in which weakened ACTH–cortisol coupling could serve as a biomarker of stress adaptation (Russell and Lightman 2019). 

### Leptin as a moderator of hypothalamic sensitivity to stress

Moderation analyses revealed that leptin influences the impact of psychological stress on gonadotropin dynamics. In the PCOM–STRESS subgroup, lower leptin levels were associated with an amplification of the suppressive effect of stress on the LH/FSH ratio, supporting its role as a contextual moderator of hypothalamic sensitivity (Evans et al. 2022; Childs et al. 2021). Positive associations between leptin, LH, and AMH further suggest that leptin helps sustain gonadotropic–granulosa coupling under stress, even though multivariate models did not identify it as a direct predictor of estradiol or AMH. This pattern suggests that leptin primarily influences reproductive function by modulating hypothalamic sensitivity to stress, rather than by directly regulating ovarian steroidogenesis (Roubos et al. 2012; Astudillo-Guerrero et al. 2025; Mélo et al. 2024).

By contrast, in the PCOM–NON-STRESS group, leptin levels were elevated but unrelated to gonadotropic output, consistent with central leptin resistance typically seen in classic PCOS, which is likely mediated by hyperinsulinemia or low-grade inflammation (Lian et al. 2016; Spritzer et al. 2015; Laughlin et al. 1997; Peng et al. 2022 and Zheng et al. 2017). Interestingly, only in this group did leptin correlate with psychological stress, suggesting that stress-related changes in leptin become more apparent when hypothalamic suppression is absent. No such associations were observed in the controls, reinforcing the view that leptin reflects stress burden specifically in neuroendocrine-sensitive phenotypes (Bouillon-Minois et al. 2021; Mélo et al. 2024). Taken together, these findings position leptin less as a direct effector of ovarian steroidogenesis and more as a contextual moderator of hypothalamic–pituitary sensitivity to stress, with distinct implications across PCOM phenotypes.

### Diagnostic and clinical implications of divergent PCOM phenotypes

These findings outline prototypical profiles for each OA subgroup. The PCOM–NON-STRESS group closely mirrors classical PCOS, characterized by elevated LH/FSH ratios, higher AMH, and leptin-related metabolic features, but without evidence of stress-induced hypothalamic suppression (Beitl et al. 2022). By contrast, the PCOM–STRESS subgroup illustrates how stress modifies the typical PCOS-like profile: hypothalamic inhibition selectively lowers LH, leading to reduced AMH and estradiol despite preserved ovarian morphology (Hager et al. 2023; Carmina et al. 2016; Beitl et al. 2022). These shifts are clinically meaningful: estradiol levels in PCOM–STRESS approach those seen in FHA, while the raised LH/FSH ratio in PCOM–NON-STRESS falls within diagnostic ranges for PCOS. Functional divergence between subgroups thus becomes visible when results are interpreted against established clinical thresholds.

In a clinical setting, incorporating brief psychometric tools such as the STAI, HADS, or PSS-10 into the evaluation of women with OA and PCOM may reveal stress-related suppression that would otherwise remain undetected. Likewise, serum leptin, considered in relation to BMI, provides a practical marker of metabolic context, flagging women who are more vulnerable to stress-induced reductions in gonadotropin secretion (Peng et al. 2022 and Zheng et al. 2017). While not intended as stand-alone diagnostic tools, these low-cost and widely accessible tests can complement existing criteria and help distinguish PCOM–STRESS from PCOS phenotypes, guiding more tailored management.

To note, the NON-PCOM/NON-STRESS group represents women with ovulatory dysfunction in the absence of PCOM and without psychological stress. This heterogeneous profile does not align neatly with either PCOS or FHA and warrants further characterization.

### Diagnostic and clinical implications

The findings of this study have practical implications for the evaluation of women presenting with OA and PCOM. In routine clinical settings, diagnosis often relies heavily on morphology, which can lead to misclassification. Our results support a more multidimensional approach that can be operationalized through simple, accessible steps:

1. Baseline endocrine work-up: gonadotropins, estradiol, and AMH remain essential first-line investigations.
2. Assessment of psychological stress: Brief, validated instruments such as the STAI, HADS, or PSS-10 may uncover stress-related vulnerabilities not apparent in standard gynecological evaluations. When stress is detected, measuring ACTH and cortisol can provide additional contextual information on HPA function, even if basal levels appear normal.
3. Evaluation of metabolic context: leptin, interpreted in relation to BMI, can provide insight into whether gonadotropin suppression reflects hypothalamic sensitivity to stress or a more classical PCOS-like profile.

Taken together, these steps provide a preliminary framework for determining whether the clinical trajectory should align with conventional PCOS management, emphasizing metabolic surveillance and ovulation induction, or whether stress reduction and hypothalamic recovery should be prioritized, as in FHA-like suppression. While exploratory, this structured approach may enhance diagnostic precision and help individualize care in women with ambiguous ovulatory dysfunction.

### Limitations and future clinical directions

Several limitations should be acknowledged in our work. Psychological stress was used both for subgrouping and as a predictor, raising the possibility of circularity. Distinguishing categorical thresholds (for classification) from continuous scores (for modelling) helped mitigate, but could not eliminate, this overlap. Stress was assessed exclusively through self-report, which is vulnerable to recall and perception bias and may not fully capture dynamic neuroendocrine reactivity. Subgroup sizes were unequal, which potentially affected the robustness of between-group comparisons, despite the use of bootstrapped confidence intervals. Hormonal assays were performed at a single time point, precluding evaluation of pulsatility and temporal variability. Finally, the Stress Index remains an exploratory composite measure that requires validation before it can be applied in clinical settings.

These considerations emphasize that our findings should be regarded as exploratory and hypothesis-generating. Future research should include longitudinal and interventional designs, with larger and more diverse cohorts, to clarify causal pathways, establish the reversibility of stress-related ovarian suppression, and determine whether gonadotropin ratios and leptin can reliably index hypothalamic sensitivity to stress. Recognizing stress-induced ovarian suppression within the PCOM spectrum may ultimately bridge the diagnostic gap between PCOS and FHA, enabling more accurate subtyping and individualized care for women with ambiguous ovulatory disorders.

## Conclusion

Our findings suggest that women with PCOM exposed to chronic psychological stress may present with a pattern of functional ovarian suppression, characterized by lower LH, estradiol, and AMH levels despite preserved morphology, compared to PCOM-NON-STRESS. These associations are consistent with stress-induced hypothalamic inhibition; however, the cross-sectional design precludes causal inference.

Brief stress questionnaires and routine LH/FSH ratios are already accessible in clinical practice, while leptin assays are feasible with standard laboratory platforms. Incorporating these low-cost measures could help identify FHA-like suppression in women otherwise meeting PCOS criteria.

Future research should prioritize longitudinal and interventional studies to confirm reversibility, clarify mechanisms, and validate leptin and gonadotropin ratios as biomarkers of hypothalamic sensitivity. Such work will be essential for refining diagnostic frameworks and improving individuzlized care in women with OA-PCOM.

## Supplementary Information

Below is the link to the electronic supplementary material.

Supplementary File 1 (DOCX 40.0 KB)

## Data Availability

The datasets generated and analysed during the current study are not publicly available due to participant confidentiality; however, they are available from the corresponding author upon reasonable request.
