# Supplementary material for: Psychological stress and functional ovarian suppression in women with PCOM: an observational study of FHA-like neuroendocrine phenotypes
Source: Arch Womens Ment Health. 2026 Jan 7;29(1):10. doi: 10.1007/s00737-025-01657-z (PMC12779685; doi:10.1007/s00737-025-01657-z)
Supplement: Supplementary file 1 — (DOCX 40.0 KB) [file 737_2025_1657_MOESM1_ESM.docx]

Supplementary Material
The following tables provide additional analyses referenced in the manuscript.

## Supplementary Table S1. Correlation matrix among hormonal, metabolic, and psychological variables (Full sample)

|  | BMI | FSH | LH | Estradiol | Cortisol | AMH | Leptin | ACTH | LH/FSH | Stress Index | AMH_NFBO |
| --- | --- | --- | --- | --- | --- | --- | --- | --- | --- | --- | --- |
| BMI | 1 |  |  |  |  |  |  |  |  |  |  |
| FSH | -0.011 (p=0.901) | 1 |  |  |  |  |  |  |  |  |  |
| LH | 0.024 (p=0.785) | 0.421 (p=0.000) | 1 |  |  |  |  |  |  |  |  |
| Estradiol | -0.032 (p=0.718) | 0.179 (p=0.038) | 0.261 (p=0.002) | 1 |  |  |  |  |  |  |  |
| Cortisol | 0.006 (p=0.949) | 0.079 (p=0.364) | -0.043 (p=0.626) | -0.129 (p=0.139) | 1 |  |  |  |  |  |  |
| AMH | 0.133 (p=0.127) | -0.024 (p=0.785) | 0.514 (p=0.000) | 0.207 (p=0.017) | -0.114 (p=0.189) | 1 |  |  |  |  |  |
| Leptin | 0.624 (p=0.000) | 0.158 (p=0.071) | 0.231 (p=0.008) | 0.191 (p=0.029) | 0.034 (p=0.702) | 0.304 (p=0.000) | 1 |  |  |  |  |
| ACTH | 0.155 (p=0.076) | 0.103 (p=0.239) | 0.019 (p=0.829) | -0.079 (p=0.366) | 0.459 (p=0.000) | -0.102 (p=0.246) | 0.175 (p=0.047) | 1 |  |  |  |
| LH/FSH | 0.057 (p=0.511) | 0.126 (p=0.146) | 0.913 (p=0.000) | 0.234 (p=0.006) | -0.081 (p=0.353) | 0.600 (p=0.000) | 0.265 (p=0.002) | 0.015 (p=0.868) | 1 |  |  |
| Stress Index | 0.215 (p=0.012) | -0.038 (p=0.662) | -0.061 (p=0.486) | -0.073 (p=0.399) | 0.020 (p=0.820) | 0.007 (p=0.940) | 0.235 (p=0.007) | 0.010 (p=0.914) | -0.075 (p=0.388) | 1 |  |
| AMH- FNBO | -0.076 (p=0.393) | 0.027 (p=0.757) | 0.236 (p=0.007) | -0.022 (p=0.803) | -0.052 (p=0.560) | 0.583 (p=0.000) | 0.078 (p=0.382) | -0.156 (p=0.078) | 0.259 (p=0.003) | 0.028 (p=0.751) | 1 |

Pearson correlation coefficients (r) and two-tailed p-values for associations between hormonal, metabolic, and psychometric variables in the full sample. Only the lower triangle is displayed for clarity.

## Supplementary Table S2 - Linear regression predicting AMH and estradiol levels (PCOM–STRESS subgroup)

## Bootstrapped estimates with 5,000 samples (BCa intervals).

| Predictor | B (AMH) | Boot p (AMH) | 95% CI BCa (AMH) | B (E2) | Boot p (E2) | 95% CI BCa (E2) |
| --- | --- | --- | --- | --- | --- | --- |
| (Constant) | 7.989 | <.0001 | [6.462, 9.556] | 53.342 | <0.001 | [42.49, 64.63] |
| LH/FSH Ratio | 2.861 | 0.048 | [0.402, 6.107] | 11.867 | 0.028 | [2.20, 24.62] |
| Leptin | 0.106 | 0.047 | [0.001, 0.199] | 0.253 | 0.305 | [–0.271, 0.754] |
| Stress Index | 0.034 | 0.441 | [–0.051, 0.134] | –0.026 | 0.910 | [–0.493, 0.404] |
| BMI | –0.068 | 0.583 | [–0.315, 0.149] | –0.380 | 0.598 | [–2.036, 1.450] |
| Stress × Leptin | –0.003 | 0.196 | [–0.009, 0.004] | –0.018 | 0.192 | [-0.409, 0.011] |

Multiple linear regression models predicting AMH and estradiol levels in the PCOM–STRESS subgroup. Predictors include LH/FSH ratio, leptin, Stress Index, BMI, and the interaction term Stress × Leptin. Bootstrapped 95% bias-corrected and accelerated (BCa) confidence intervals were computed using 5,000 samples. All predictors were mean-centered.

# Supplementary Table S2a - Summary of mediation and moderation pathways tested in the PCOM–STRESS subgroup.

| Path tested | B / β | 95% CI | p | Outcome | Clinical note |
| --- | --- | --- | --- | --- | --- |
| Stress Index → LH/FSH | –0.33 | — | .027 | Significant | Higher stress linked to reduced LH/FSH (FHA-like) |
| LH/FSH → Estradiol | +11.87 | [2.20, 24.62] | .028 | Significant | Lower LH/FSH predicts lower estradiol (↓ granulosa stimulation) |
| LH/FSH → AMH | +2.86 | [0.40, 6.11] | .048 | Significant | Reduced LH/FSH associated with diminished AMH (↓ granulosa–theca support) |
| Stress Index → Estradiol (direct) | — | — | n.s. | Not significant | No independent stress effect beyond LH/FSH |
| Stress Index → AMH (direct) | — | — | n.s. | Not significant | No independent stress effect beyond LH/FSH |
| Indirect: Stress → LH/FSH → Estradiol | –0.185 | [–0.397, –0.042] | — | Significant mediation | Stress reduces estradiol indirectly via LH/FSH suppression |
| Indirect: Stress → LH/FSH → AMH | –0.051 | [–0.104, –0.015] | — | Significant mediation | Stress reduces AMH indirectly via LH/FSH suppression |
| Leptin → AMH | +0.106 | [0.001, 0.199] | .047 | Significant | Leptin supports AMH production (permissive signal) |
| Leptin → Estradiol | +0.253 | [–0.271, 0.754] | .305 | Not significant | No evidence of direct leptin effect on estradiol |
| BMI → AMH / Estradiol | — | — | n.s. | Not significant | BMI not independently predictive in this subgroup |
| Interaction: Stress × Leptin → LH/FSH | –0.001 | [–0.001, –6.04×10⁻⁵] | .004 | Significant interaction | Low leptin amplifies stress-related LH/FSH suppression |

Note. B = unstandardized coefficient; β = standardized coefficient; CI = confidence interval; n.s. = not significant. Significant effects are highlighted as either direct, indirect (mediation), or interaction terms.
